# Lubricating conditions: effects on friction between orthodontic brackets and archwires with different cross-sections

**DOI:** 10.1590/2177-6709.24.2.066-072.oar

**Published:** 2019

**Authors:** Fabrício Anderson Carvalho Almeida, Anna Paula Costa Ponte Sousa Carvalho Almeida, Flávia Lucisano Botelho Amaral, Roberta Tarkany Basting, Fabiana Mantovani Gomes França, Cecilia Pedroso Turssi

**Affiliations:** 1 Faculdade São Leopoldo Mandic, Instituto de Pesquisas São Leopoldo Mandic, Divisão de Ortodontia (Campinas/SP, Brazil).; 2 Universidade Federal do Pará, Divisão de Radiologia (Belém/PA, Brazil).; 3 Faculdade São Leopoldo Mandic, Instituto de Pesquisas São Leopoldo Mandic, Divisão de Cariologia e Odontologia Restauradora (Campinas/SP, Brazil).

**Keywords:** Lubrication, Friction, Orthodontic wire

## Abstract

**Objective:**

This study investigated the effect of the condition of lubrication on the friction between brackets and NiTi archwires of different rounded cross-sections.

**Methods:**

Brackets (Roth, GAC) were affixed to a device connected to a universal testing machine into which segments of archwire were placed (NiTi, Nitinol, GAC) with cross-sections of 0.012-in, 0.016-in and 0.020-in. Once the wire was in the bracket slot, the following lubricants were applied: human saliva (HS: positive control), distilled water (DI), mucin-based (MUC) or carboxymethylcellulose-based (CMC) artificial saliva. In the negative control group, no lubricant was used. The combination between the wire cross-sections and the lubrication condition generated 15 groups with 15 samples each. Data were submitted to two-way analysis of variance and Tukey’s test.

**Results:**

There was no significant interaction between the wire cross-section and the condition of lubrication (*p*= 0.901). Irrespective of whether lubricants were used or not, there was a significant increase in friction with an increase in the cross-section of the wire (*p*< 0.001). For any wire, the group tested in the presence of MUC was not different from that in which HS was applied. On the other hand, when the application of lubricants was suppressed, significantly higher friction values were observed. The CMC group and the DI group demonstrated intermediate behavior.

**Conclusions:**

Friction increased with the increase of the cross-section of the NiTi archwire, but regardless of the archwire, friction with MUC artificial saliva was similar to that of HS and lower than in dry conditions.

## Resumo

**Objetivo:**

este estudo investigou o efeito das condições de lubrificação no atrito entre braquetes e fios de NiTi de diferentes secções transversais.

**Métodos:**

os braquetes (Roth, GAC) foram fixados a um dispositivo conectado a uma máquina de ensaio universal, que recebeu segmentos de fio de NiTi (Nitinol, GAC) com secções de 0,012”; 0,016” ou 0,020”. Após o fio ter sido instalado no *slot* do braquete, aplicou-se um dos seguintes lubrificantes: saliva humana (SH: controle positivo), água destilada (AD) ou saliva artificial à base de mucina (MUC) ou de carboximetilcelulose (CMC). No grupo controle negativo, nenhum lubrificante foi utilizado. As combinações entre as secções dos fios e as condições de lubrificação geraram 15 grupos, com 15 amostras cada. Os dados foram submetidos à análise de variância a dois critérios e ao teste de Tukey.

**Resultados:**

não houve interação significativa entre a secção do fio e a condição de lubrificação (*p* = 0,901). Independentemente de ter sido utilizado ou não lubrificante, observou-se elevação significativa do atrito com o aumento da secção transversal do fio (*p* < 0,001). Para qualquer um dos fios, o grupo testado na presença de MUC não diferiu daquele em que se aplicou SH. Por outro lado, quando suprimiu-se os lubrificantes, constatou-se atrito significativamente mais elevado. Os grupos CMC e AD demonstraram comportamento intermediário.

**Conclusões:**

o atrito se elevou com o aumento da secção transversal dos fios de NiTi, mas a despeito do fio, o atrito com a saliva à base de MUC foi similar àquele com a SH e menor do que sob condição a seco.

## INTRODUCTION

During the mechanical therapy, the friction between the bracket-archwire interface could prevent the action of forces required for a particular movement.^1^ Studies demonstrated that approximately 12 to 60% of the force used to move a tooth is dissipated in the form of friction.^2^ ^,^ ^3^ Consequently, a delay could occur in the biological response to orthodontic movement.^4^

The most important factors that may have an impact on friction are: the composition of the bracket; the archwire alloy; the cross-sectional size of the archwire; the type of ligation system and the surface roughness of the bracket-archwire assembly.^5^ ^-^ ^11^ Specifically with regard to the cross-sectional size of the archwire, some authors reported that friction in brackets augments with increased size of rounded wire cross-section.^6^ ^,^ ^8^

In addition to the factors related to the orthodontic appliances, saliva is considered to be a biological variable associated with friction, as it acts as a lubricant during sliding mechanics.^12^ This fact should be taken into account in laboratory studies that aim to evaluate the performance of the archwire-bracket combinations. However, in the majority of the research studies, the friction test has been conducted without the use of any lubricant,^6^ ^,^ ^8^ ^,^ ^13^ ^-^ ^15^ which does not represent the clinical reality where there is saliva introduced during the movement of the archwire on the bracket. To remedy this situation, distilled water has been used as a lubricant.^16^ Although in this case the test is conducted in the presence of a lubricant, water does not have the lubricating ability of natural human saliva.^17^ ^,^ ^18^

Although human saliva could be considered the best fluid to use, studies have demonstrated conflicting results with regard to its lubricating capacity.^17^ ^,^ ^19^ ^,^ ^20^ Therefore, a suitable alternative would be the use of artificial saliva. However, in order to simulate the effects that human saliva would provide clinically, it is essential that artificial saliva has similar rheological properties to those of human saliva. 

Despite this requirement, in some studies where artificial saliva has been introduced during the friction test,^9^ ^,^ ^10^ ^,^ ^21^ ^-^ ^23^ no mention has been made regarding the ability of these fluids to simulate the viscosity and adsorption of human saliva. Exceptions are the investigations carried out by Al-Mansouri et al^17^ and Leal et al.^18^ However, while in the latter study artificial saliva was found to be a suitable substitute for human saliva in friction tests; in the former, artificial saliva was not considered an ideal alternative to human saliva. Such discrepant findings may be in part explained by the different types of brackets used and by the cross-sectional size of the archwires tested. 

Given that the wire cross-section is important in the context of friction,^6^ ^,^ ^8^ but that the knowledge in this area has been generated from tests carried out under friction conditions that may not approximate clinical conditions, the present study analyzed the effect of lubricating conditions on the friction between brackets and NiTi archwires of varying cross-sectional sizes. 

## MATERIAL AND METHODS

### Experimental design

**Figure 1:**
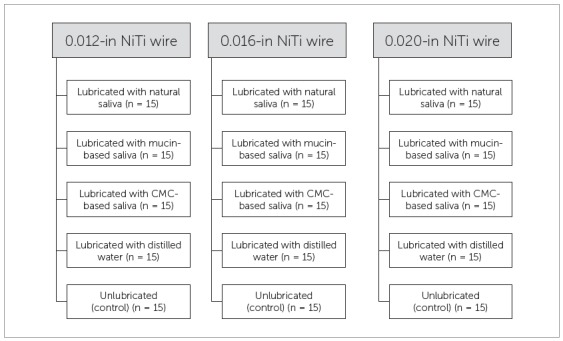
Schematic presentation of the experimental layout.

This study consisted of a completely randomized design, with a 3x5 factorial arrangement. The factors studied were Cross-section of NiTi Archwire, at three levels (0.012-in, 0.016-in and 0.020-in) (Table 1) and Lubricant, at five levels (no lubricant, natural human saliva, distilled water, mucin-based artificial saliva, carboxymethylcellulose-based artificial saliva) (Fig 1). The combination between the levels of both factors generated 15 groups with 15 samples each. The response variable was friction, measured in Newtons (N). 

**Table 1:** Characterization of the archwires tested.

Archwire	Brand	Lot number
0.012-in	Nitinol, Dentsply GAC, Bohemia, NY, USA	088243
0.016-in	Nitinol, Dentsply GAC, Bohemia, NY, USA	088649
0.020-in	Nitinol, Dentsply GAC, Bohemia, NY, USA	059495

### Ethical aspects 

After the approval by the local Research Ethics Committee of the Faculty of Dentistry and Center of Dental Research São Leopoldo Mandic (protocol #0510), a subject signed the free and informed consent form and took part as the only donor of non-stimulated saliva. The criteria used to select this participant were as follows: normal saliva flow and non-use of medication; no need for dental treatment, as well as fixed or removable prostheses or orthodontic appliances; absence of systemic diseases, tobacco use, pregnancy, lactation and alcoholism.

### Collection of natural human saliva and acquisition of artificial saliva

All samples of non-stimulated human saliva were collected from a female donor in the morning, at least 2 hours after eating and brushing the teeth. The collections took place immediately prior to the friction tests. The saliva was expelled into a funnel positioned over a sterile test tube and packed with ice in a compartment, awaiting use. Both mucin and carboxymethylcellulose-based artificial salivas were prepared according to the formulation proposed elsewhere.^24^ The mucin-based preparation was composed of porcine mucin (3.5 g), xylitol (2 g), methylparaben (100 mg), EDTA (50 mg), benzalkonium chloride (2 mg), and sodium fluoride (0.42 mg) in 100 mL of aqueous solution. The carboxymethylcellulose-based saliva was composed of carboxymethylcellulose (500 mg), sodium fluoride (20 mg), xylitol (3 g), potassium phosphate (35 mg), sodium chloride (90 mg), and potassium chloride (120 mg) in 100 mL of aqueous solution. 

### Friction testing

For the friction test, the NiTi archwires were cut into 3-cm segments with a cutting plier, which provided 75 samples for each of the three types of wire tested. 

**Figure 2:**
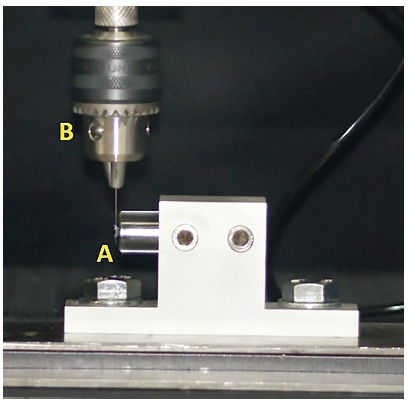
Acrylic cylindrical base with the bonded bracket fixed on the universal testing machine (A), whose moving upper arm had a clamp device (B) to firmly attach the wires.

**Figure 3:**
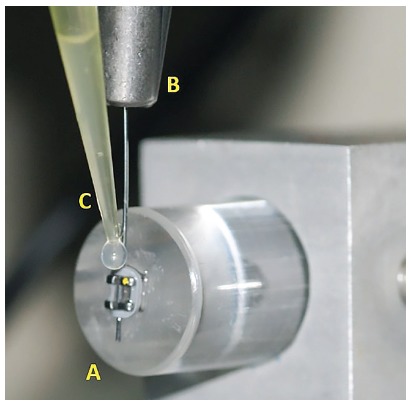
Close view of the experimental apparatus, showing: the wire/bracket/ligature bonded to the acrylic cylindrical base (A), the clamp device (B) and the lubricant application (C).

Each bracket (Roth, Dentsply GAC, Bohemia, NY, USA) was bonded to an acrylic cylindrical base using a cyanoacrylate-based adhesive (Super Bonder, Loctite-Henkel, São Paulo/SP, Brazil) and then this ensemble (Figs 2A and 3A) was firmly fixed on the universal testing machine (EMIC DL 10000, São José dos Pinhais/PR, Brazil). 

According to a random sequence, a NiTi wire segment was inserted into the clamp of a device connected to the moving upper arm of the universal testing machine with 20 N load cell and into the bracket slot (Figs 2B and 3B). An elastomeric ligature was placed over the bracket tie wings engaging the wire. Therefore, one end of the tested wire was left free, and the other end was firmly attached to the moving upper arm of the universal testing machine. Care was taken to avoid introducing torsion into the tested wire.

In the groups assigned for testing in the presence of natural human saliva, artificial saliva or distilled water, these lubricants were applied to the wire, close to the bracket slot, with the aid of a micropipette (Fig 3C), in a standard volume of 50 µL. In the negative control group, the test was carried out under dry friction, i.e. without any lubricant.

The universal testing machine was used for measuring the generated frictional force at the bracket-wire interface by sliding the wire through the bracket slot under a 1-mm tangential displacement. The friction testing was based on the classical model of friction and did not include binding or notching. The tested wire was pulled upward through the bracket slot at a speed of 3 mm/min, eight consecutive times. Before testing, the reading was set to give a zero reading after the wire was lightly tightened and was completely in a straight, vertical position toward the moving arm. This ensured that the force transmitted by the moving arm to the wire/bracket/ligature assembly was from friction-only origin. 

The frictional force generated by each wire/bracket/ligature assembly during the pulling upward movement was registered in Newtons (N) by the tension load cell, and the eight consecutive values obtained for a given wire segment were averaged to be used as the outcome value. New bracket and ligature were used for each one of the 15 repetitions per group.

### Statistical analysis

The friction values were subjected to two-way analysis of variance and to Tukey’s test, at a significance level of 0.05. The statistical calculations were carried out using the SPSS 20 software (SPSS Inc., Chicago, IL, USA).

## RESULTS

As presented in Table 2, the two-way analysis of variance showed that there was no significant interaction between the wire cross-section and the lubrication condition (*p*= 0.901). Regardless of the usage of lubricants, there was a significant increase in friction with the increase in the cross-section of the NiTi wire (*p*< 0.001). Irrespective of the cross-section of the NiTi wire, the lubrication condition has an impact on friction (*p*= 0.009). The Tukey’s test revealed that, for any wire, the group tested in the presence of mucin-based saliva did not differed from the one where human saliva was applied (Table 3). On the other hand, when the application of lubricants was suppressed, significantly higher friction values were observed (Table 3). The groups whose wire-bracket combinations were tested in the presence of carboxymethylcellulose-based saliva and distilled water presented intermediate behavior (Table 3).

**Table 2:** Results of the two-way analysis of variance.

Source	SQ	DF	Mean square	F	p value
Wire	74.74	2	37.37	75.52	0.000
Lubricant	6.89	4	1.72	3.48	0.009
Wire x lubricant	1.71	8	0.21	0.43	0.901
Error	103.92	210	0.49		
Total	187.26	224			

SQ = sum of squares; DF = degrees of freedom.

**Table 3:** Mean ± standard deviations values (95% confidence interval) of the friction (in N) by lubrication condition and orthodontic wire cross-section.

Lubricant	Orthodontic wire cross-section			Overall mean
	0.012-in	0.016-in	0.020-in	
Natural saliva	1.45 ± 0.70 (1.06 - 1.84)	1.77 ± 0.48 (1.50 - 2.03)	2.81 ± 0.67 (2.45 - 3.19)	2.01 ± 0.85^a^ (1.76 - 2.27)
Mucin-based saliva	1.52 ± 0.35 (1.33 - 1.72)	1.87 ± 0.60 (1.54 - 2.20)	2.83 ± 0.83 (2.37 - 3.29)	2.08 ± 0.83^a^ (1.83 - 2.32)
CMC-based saliva	1.69 ± 0.32 (1.51 - 1.86)	1.98 ± 0.40 (1.76 - 2.20)	2.96 ± 0.61 (2.62 - 3.30)	2.21 ± 0.71^ab^ (2.00 - 2.42)
Distilled water	1.76 ± 0.54 (1.46 - 2.06)	2.20 ± 0.77 (1.77 - 2.63)	2.93 ± 0.68 (2.56 - 3.31)	2.30 ± 0.82^ab^ (2.05 - 2.54)
None (control)	1.72 ± 0.61 (1.39 - 2.07)	2.33 ± 1.08 (1.73 - 2.92)	3.47 ± 1.26 (2.77 - 4.16)	2.51 ± 1.23^b^ (2.14 - 2.88)
Overall mean	1.63 ± 0.53^A^ (1.51 - 1.75)	2.03 ± 0.72^B^ (1.86 - 2.19)	2.98 ± 0.85^C^ (2.81 - 3.20)	___

CMC= carboxymethylcellulose. Different uppercase letters indicate significant difference between the wire cross-sections. Different lowercase letters indicate significant difference between the lubricants.

## DISCUSSION

Although the major advantage of laboratory studies is to allow control of experimental conditions, it is important that the knowledge acquired during friction testing does not stray too far from clinical reality. To this end, the use of lubricants can be considered of utmost importance as, *in vivo*, saliva acts as a lubricant. The literature, however, makes more reference to testing under dry friction conditions.^6^ ^-^ ^8^ ^,^ ^13^ ^-^ ^15^ Despite the fact that some researchers have used artificial saliva,^9^ ^,^ ^10^ ^,^ ^21^ ^,^ ^22^ ^,^ ^23^ in almost all of these studies, no attention was directed toward the rheological characteristics of the artificial saliva used. Based on that and considering that the cross-section of orthodontic wires is also a factor that influences sliding mechanics, in this study it was evaluated the friction established between orthodontic brackets and NiTi archwires with varying cross-sections, under different lubrication conditions. 

In the current study, rounded NiTi wires were used, with cross-sections of 0.012-in; 0.016-in and 0.020-in, and it was found that as the cross-sections increased, a progressive increase in friction occurred, both in dry state and in the presence of lubricants. Such result seems to indicate that apart from lubrication, the cross-section size of the archwire plays an important role in the sliding mechanism, as reported in previous investigations.^6^ ^,^ ^8^

Regardless the archwire cross-section, there was no difference in friction when tests were run in the presence of artificial saliva and natural human saliva. These data corroborate early observations made by Leal et al,^18^ who compared the effect of the same lubricants used herein on the friction between CuNiTi wires positioned in the slots of self-ligating brackets. In the quoted paper, the authors attributed the lack of differences between human and artificial saliva to the capacity of the latter to adsorb and form film. 

Despite the fact that no difference was noticed between the mucin- or carboxymethylcellulose-based salivas in the current study, only the mucin formulation provided statistically significant less friction than the dry condition, making the mucin-based saliva the preferable lubricant. Such recommendation seems even more pertinent if one considers that the mucin-based saliva used has a viscoelasticity similar to that of human saliva.^24^ In fact, the mucin-based saliva used herein has been considered the best option for substituting natural saliva.^24^ ^-^ ^26^

Contrary to the present results, in a previous study, friction generated by an artificial saliva was greater than that of human saliva, water and dry friction.^19^ This may be explained by the fact that artificial saliva has rheological properties that limit the formation of film. In another paper,^27^ in which artificial saliva caused increased friction, brackets were angled, a condition that considerable increases friction and presumably makes lubrication of secondary importance. 

Aligned with other studies,^18^ ^,^ ^21^ in this investigation friction was higher in the absence of any lubricant. Under dry condition, it is assumed that throughout the eight sliding movements repeated in each wire/bracket/ligature assembly, the wire and bracket asperities intimately contacted each other, probably leading to a minimal formation of debris and increased friction.

## CONCLUSION

Friction increased with the increase of the cross-section of the NiTi archwire, but during testing, regardless of the archwire, friction with mucin-based artificial saliva was similar to that of natural human saliva and lower than under dry conditions.
